# Elimination of the Femoral Neck in Measuring Femoral Version Allows for Less Variance in Interobserver Reliability

**DOI:** 10.3390/medicina57121363

**Published:** 2021-12-14

**Authors:** Radomir Dimovski, Robert Teitge, Nicholas Bolz, Patrick Schafer, Vamsy Bobba, Rahul Vaidya

**Affiliations:** Detroit Medical Center, Department of Orthopaedic Surgery, Wayne State University, Detroit, MI 48201, USA; rteitge@dmc.org (R.T.); nbolz2@dmc.org (N.B.); pschafer@dmc.org (P.S.); vbobba@dmc.org (V.B.); rvaidya@dmc.org (R.V.)

**Keywords:** femoral version, femoral osteotomy, femoral malunion

## Abstract

*Background and Objectives*: Producing consistent measures of femoral version amongst observers are necessary to allow for an assessment of version for possible corrective procedures. The purpose of this study was to compare two computed tomography (CT)-based techniques for the reliability of measuring femoral version amongst observers. *Materials and Methods*: Review was performed for 15 patients post-femoral nailing for comminuted (Winquist III and IV) femoral shaft fractures where CT scanograms were obtained. Two CT-based techniques were utilized to measure femoral version by five observers. *Results*: The mean femoral version, when utilizing a proximal line drawn down the center of the femoral head-neck through CT, was 9.50 ± 4.82°, while the method utilizing the head and shaft at lesser trochanter centers produced a mean version of 18.73 ± 2.69°. A significant difference was noted between these two (*p* ≤ 0.001). The method of measuring in the center of the femoral head and neck produced an intraclass correlation coefficient (ICC) of 0.960 with a 95% confidence interval lower bound of 0.909 and upper bound of 0.982. For the method assessing version via the center of the head and shaft at the lesser trochanter region, the ICC was 0.993 with a 95% confidence interval lower bound of 0.987 and an upper bound of 0.996. *Conclusions*: The method of measuring version proximally through a CT image of the femoral head–neck versus overlaying the femoral head with the femoral shaft at the most prominent aspect of the lesser trochanter produces differing version measurements by roughly 10° while yielding an almost perfect interobserver reliability in the new technique. Both techniques result in significantly high interobserver reliability.

## 1. Introduction

Femoral version is the angular difference of the proximal femur relative to the distal femur in the axial plane. Defining the ideal method for the assessment of femoral version has long been contemplated in the orthopedic literature. Producing repeatable measures of femoral version are necessary to allow for assessment of torsional abnormalities for possible corrective procedures.

In 1948, Kingsley et al. evaluated cadaveric femurs and noted that when assessing femoral version, as described by the methods of Pearson and Bell, the femoral head was displaced anteriorly or posteriorly in 68.7% of femurs relative to the true longitudinal axis of the neck of the femur. Kingsley’s assessment of femoral version involved placing the cadaveric femur on a flat tabletop and measuring the angle produced by the longitudinal axis of the femoral neck in reference to the flat tabletop. They argued at this time to not use any point in the head but instead just two points within the axis of the neck for torsional assessment [1]. In their study of 630 cadaveric femurs, both pediatric and adult, they found the normal range of version to be from 20°of retroversion to 38° of anteversion with an average of about 8° of anteversion. In 1954, Billing defined femoral version as the angle in the transverse plane between the axis of the femoral neck, as described by the center of the femoral head to the center of the femoral neck base in reference to the condylar axis, which he describes as parallel to the posterior femoral condyles [2]. 

In the 1980s, the use of computed tomography (CT) was popularized as the method of choice for assessing femoral version. In comparison to Billing’s utilization of a single cut of the head–neck axis, Murphy utilized two axial cuts of femurs set in plaster to mimic CT scans about the femoral neck to generate the proximal axis with points from the center of the femoral head to the center of the femoral diaphysis at the base of the femoral neck. Murphy found that his method resulted in average femoral anteversion which differed from a method involving the femoral neck axis [3]. A wide range of femoral versions have been published from 23° of retroversion to 48° of anteversion via multiple measurement modalities [1,4,5,6,7,8,9,10,11,12].

Producing an accurate and reliable method of measuring version has long been sought with multiple methods previously assessed [3,11,13,14,15,16]. The use of varying measurement techniques has resulted in a wide range of normal for femoral version. Currently, variants of version measurement are conducted on several software platforms and there is no consensus. Many methods have utilized cadaveric femurs isolated from their host to mimic CT images and approximate version measurements while showing high levels of intraobserver and interobserver agreement [16]. However, in live patients, we are relegated to using CT scan slices. This is the first study assessing version with the femoral shaft at the level of the lesser trochanter as a reference point. We chose this point as it is easily identified, eliminates capito-collar differences as noted by Kingsley, and Murphy’s work showed small variance with the incorporation of the centroid of the femoral shaft. The purpose of this study is to determine if eliminating the femoral neck as a reference point in the assessment of femoral version while maintaining the posterior condyle axis as the distal reference would lead to higher interobserver reliability. 

## 2. Materials and Methods

**Figure 1:**
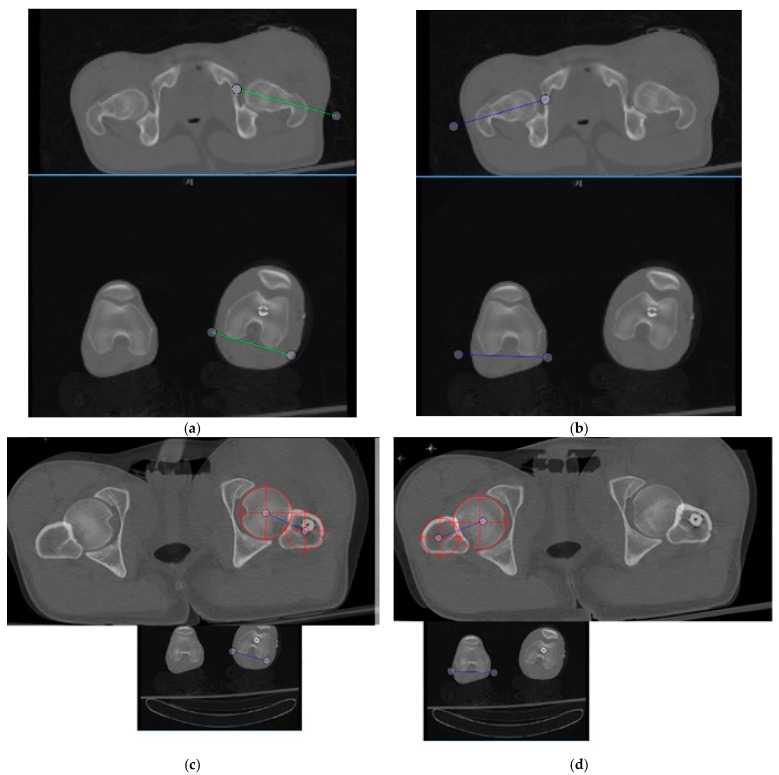
Shown above are the measurements of a patient’s left femur (**a**) and right femur (**b**) utilizing a line centered through the femoral head–neck axis as the proximal reference (Method 1). (**c**) shows the left femur measurement of the same patient using the hip center tool for the femoral head and shaft at the most prominent aspect of the lesser trochanter while (**d**) shows the same technique utilized for the right femur (Method 2). These measurements are screenshots from the Bonesetter application.

An internal review board (IRB) approved retrospective study was performed on 15 patients post-femoral nailing for comminuted (Winquist III and IV) femoral shaft fractures, where computed tomography scanograms were obtained. Bilateral femurs from each of the 15 patients, 30 femurs total, were used and axial CT images were taken at the level best visualizing the femoral head–neck (Method 1) as well as of the center of the femoral head and the most prominent aspect of the lesser trochanter distally (Method 2). The most prominent aspect of the lesser trochanter was chosen as the axial segment to overlay on the femoral head as it provides an easily reproducible image overlay and landmark. Slices were selected by referencing each patient’s CT scanogram in the axial and coronal planes with the axial cuts for head, neck, and lesser trochanter being taken at the level of greatest femoral head diameter and lesser trochanter prominence. The images of the femoral head center and shaft at the lesser trochanter were overlaid to scale within the Bonesetter application (www.detroitbonesetter.com) (Figure 1). Post-traumatic femoral version abnormalities have been analyzed previously via the use of the Bonesetter application in the assessment of second-generation femoral nailing for version and length assessments [17]. This platform allows for CT images to be overlayed. Each observer was required to overlay axial images for the widest diameter of the femoral head and most prominent aspect of the lesser trochanter. The center of the head and shaft at these respective points was assessed by each observer, and a line connecting those points was the proximal reference to measure version. Both image sets utilized the distal femoral axial cut which best displayed the posterior condylar axis for each limb separately. Femoral version was measured by 5 observers with the images displayed in random order to mask the observants from recognizing any of the 15 patients’ bilateral limbs between measurement methods. Measurements using the two methods were conducted consecutively by each observer to total 30 measurements for each method per participant when accounting for bilateral extremities.

The 5 sets of measures for both measurement methods were analyzed for the average versions measured amongst the observers along with standard deviation. These means were further assessed with a One-Sample *T*-test. Intraclass correlation coefficient (ICC) and their 95% confidence intervals were calculated based on mean rating, absolute agreement, and 2-way mixed-effects model for each method. ICCs were compared using the scoring system described by Fleiss et al. [18]. All statistics were performed on IBM SPSS Statistics 26 (IBM Corp., Armonk, NY, USA). 

## 3. Results

The mean femoral version for the method utilizing a line drawn down the center of the head–neck was 9.50 ± 4.82°, while the method utilizing the head and shaft centers produced a mean version of 18.73 ± 2.69°. Range of measurements for the Method one group, which included our patients’ axial CT cuts through the femoral head–neck–GT, means were from 20° of retroversion to 37° of anteversion. Method two resulted in a range from 23° of retroversion to 52° of anteversion. A significant difference was noted between these two means via a One-Sample *T*-test (*p* = 0.011). Since the methods produce significantly different measures of femoral version, the inter-rater reliability of each measure was checked by ICC to determine which method is best replicated across observers. Method one produced an ICC of 0.960 with a 95% confidence interval lower bound of 0.909 and an Upper bound of 0.982. For Method two, the intraclass correlation coefficient was 0.993 with a 95% confidence interval lower bound of 0.987 and an upper bound of 0.996 (Table 1).

## 4. Discussion

Our results show that the two measurement techniques resulted in significantly different femoral version averages with a mean difference of roughly 9° more anteversion in the model utilizing the femoral head to the center of the shaft at the lesser trochanter. The mean version generated by Method one and Method two fall within the previously published range of normal femoral version [4,5,6,7,8,9,10,11]. As noted by Kaiser et al., measuring femoral version with different techniques on the same femurs will elicit differences in the average version [19]. Each method can elicit a variance in standard values. In our study, the average femoral version was found to range from 20° of retroversion to 37° of anteversion for the head–neck method in comparison to 23° of retroversion to 54° of anteversion. When combining measurements of bilateral femurs for a patient, the head–neck method can result in upwards of 19.3° of difference when considering the extremes of mean standard deviation while the new studied method could produce upwards of 10.8° difference. Differences of 15° version between a patient’s femurs have been found to lead to impairment of function [20]. 

Measurements were conducted on the Bonesetter application, which removes the variable of creating a horizontal line to measure as was conducted in previous analysis utilizing cadaveric femurs [19]. This same study assessed similar measurement techniques which were proximal to the lesser trochanter, and the ICC of these was 0.96, using an extrapolation by Kaiser of Yoshioka’s suggestion to view the proximal femur as two center points in the neck from above, with the distal femoral angle being separately measured. Our study found that when combining the proximal and distal femoral measurements as the centers of head to the shaft at the lesser trochanter and the posterior condylar axis of the distal femur, we were able to achieve a 95% confidence interval greater than 0.987, which is better than all previous methods. Although there is a difference in the version measurement, both methods allow for excellent interobserver reliability, with the new method displaying the highest reported intraclass reliability noted in literature for version measurements. This was comparable to observers measuring just the posterior condylar axis alone in the Kaiser et al. study. This indicates that although the two methods produce differing version measurements, the new method allows for the best reproducibility of studied methods amongst multiple observers. This may translate to more reproducible version measures for surgeons prior to surgical intervention for correction limb rotational deformities.

One limitation to our study is the use of postoperative fracture patients. This would allow for a greater range of measurements per patient which may deviate from previously published normal femoral version values due to the version created during an operative intervention. We also did not compare to multiple other measurement methods but rather compared it to how we have traditionally measured femoral version in our institution.

## 5. Conclusions

In conclusion, we found that utilizing an overlay of the center of the femoral head and the center of the femoral shaft at the most prominent aspect of the lesser trochanter for the proximal reference of femoral version with the posterior condylar axis as the distal reference allows for the highest interobserver reliability noted to date. 

## Figures and Tables

**Table 1:** Results.

Measurement Technique	Mean Femoral Version *	Range of Version	ICC
Method 1	9.50 ± 4.82°	−20° to 37°	0.960 (95% CI 0.909–0.982)
Method 2	18.73 ± 2.69°	−23° to 52°	0.993 (95% CI 0.987–0.996)

* Indicates significant difference in mean femoral version; ICC = intraclass correlation coefficient.

## Data Availability

The data presented in this study are available on request from the corresponding author. The data are not publicly available due to restrictions of our institutional database and restricting the data to remain HIPAA compliant.
